# miR-155 as an Important Regulator of Multiple Sclerosis Pathogenesis. A Review

**DOI:** 10.3390/ijms22094332

**Published:** 2021-04-21

**Authors:** Karina Maciak, Angela Dziedzic, Elzbieta Miller, Joanna Saluk-Bijak

**Affiliations:** 1 Department of General Biochemistry, Faculty of Biology and Environmental Protection, University of Lodz, Pomorska 141/143, 90-236 Lodz, Poland; karina.maciak@edu.uni.lodz.pl (K.M.); joanna.saluk@biol.uni.lodz.pl (J.S.-B.); 2 Department of Neurological Rehabilitation, Medical University of Lodz, Milionowa 14, 93-113 Lodz, Poland; elzbieta.dorota.miller@umed.lodz.pl

**Keywords:** miR-155, microRNA (miRNA), neuroinflammation, multiple sclerosis (MS), autoimmunity, biomarkers

## Abstract

Multiple sclerosis (MS) is a chronic, immune-mediated disease and the leading cause of disability among young adults. MicroRNAs (miRNAs) are involved in the post-transcriptional regulation of gene expression. Of them, miR-155 is a crucial regulator of inflammation and plays a role in modulating the autoimmune response in MS. miR-155 is involved in blood–brain barrier (BBB) disruption via down-regulation of key junctional proteins under inflammatory conditions. It drives demyelination processes by contributing to, e.g., microglial activation, polarization of astrocytes, and down-regulation of CD47 protein and affecting crucial transcription factors. miR-155 has a huge impact on the development of neuropathic pain and indirectly influences a regulatory T (Treg) cell differentiation involved in the alleviation of pain hypersensitivity. This review also focused on neuropsychiatric symptoms appearing as a result of disease-associated stressors, brain atrophy, and pro-inflammatory factors. Recent studies revealed the role of miR-155 in regulating anxiety, stress, inflammation in the hippocampus, and treatment-resistant depression. Inhibition of miR-155 expression was demonstrated to be effective in preventing processes involved in the pathophysiology of MS. This review aimed to support the better understanding the great role of miR-155 dysregulation in various aspects of MS pathophysiology and highlight future perspectives for this molecule.

## 1. Introduction

Multiple sclerosis (MS) is a chronic, untreatable disease of the central nervous system (CNS) and the most common cause of neurological disability diagnosed in young adults. It is estimated that the average age of sufferers globally is 32 years. This distinguishes the condition from other neurodegenerative diseases and makes it an increasing socioeconomic and medical problem. As of 2020, there were an estimated 2.8 million people diagnosed with MS worldwide; in 2013, the number was 2.3 million [1].

In MS, complex processes involving both genetic (e.g., polymorphism of HLA-DPB1 allele), epigenetic (e.g., miRNAs) and environmental (e.g., smoking, infectious diseases, vitamin D deficiency) determinants lead to autoimmunity and, consequently, demyelination, axons damage, and neurodegeneration [2,3,4,5]. However, the picture of causative factors underlying the disease remains unclear. Additionally, MS is a heterogeneous disease with a highly individualized clinical course. This makes differential diagnosis and monitoring of treatment efficacy difficult, as well as applying effective therapy without the risk of complications. There is a necessity to search for specific biochemical, molecular, and genetic factors that can reliably correlate with the disease development degree.

Numerous studies confirm a pivotal role of small (21–23 nucleotides) non-coding RNAs—microRNAs (miRNAs) as crucial regulators of biological processes associated with the pathophysiology of various conditions including cancer, autoimmune, neurodegenerative, and infectious diseases [6,7,8,9,10]. Moreover, miRNAs are likely to meet the criteria for a potentially good prognostic marker. Namely, one of the crucial characteristics of the biomarker is the ability to be detected in a non-invasive manner. Moreover, the biomarker should be specific, sensitive, and susceptible to change with disease progression or remission. The final important criterion is its applicability in the bench-to-bedside approach [11]. miRNAs are easy to obtain as they occurred in body fluids so there is no necessity to isolate them in an invasive way from cells or tissues. These molecules could be stored for a long period, subjected to freezing and thawing, and are resistant to pH extremes. Moreover, blood miRNAs show resistance to ribonucleases and have an average half-life of 5 days, as shown in one study [10,11].

Available data identify miR-155 as so-called inflamma-miR, a powerful activator of the inflammation, the essential miR in the autoimmune diseases’ pathogenesis because of its influence on myeloid cell polarization to a phenotypic and functional pro-inflammatory form [12,13]. Additionally, miR-155 dysregulation has a role in many human cancers, hematological malignancies, and reactions to viral infections [14]. miR-155 can serve as a significant player in the pathogenesis of MS. Successfully investigating how miR-155 contributes to MS may provide innovative determinants of neurodegeneration with the great potential for future use in diagnostics.

miR-155 activity in peripheral immune cells and brain resident cells in the context of MS was analyzed in detail in a review by McCoy [15]. This paper aims to consolidate the results of studies reflecting the consequences of miR-155 activity in certain elements of the pathophysiology of MS. Collecting the emerging data on this topic may contribute to increase understanding of the miR-155 operation and emphasize the potential of this molecule to be used in future research toward advancing the diagnosis and treatment of MS. This review will initially focus on summarizing the role of miR-155 in the context of two phenomena underlying the initiation of the MS disease process—blood–brain barrier (BBB) damage and demyelination. Second, an attempt will be made to present the available studies shedding light on miR-155 as a relevant element of the pathophysiology of neuropathic pain and cognitive impairment, which are serious dysfunctions of the complex clinical symptomatology of MS.

## 2. Multiple Sclerosis

Clinically, several subtypes of MS can be distinguished. The most prevalent form is relapsing-remitting multiple sclerosis (RRMS), characterized by periods of flares and silencing (remission). In most cases, as a result of accumulating neurological injuries, secondary progressive multiple sclerosis (SPMS) develops, and recovery phases are no longer observed. A constant progression of nervous system symptoms and occasional phases of stabilization or remission are characteristic of primary progressive multiple sclerosis (PPMS). Progressive-relapsing multiple sclerosis (PRMS) is observed when the disease progression is continuous and acute throwing episodes occur regularly [16,17].

The pathophysiology of MS is based on a persistent inflammatory state involving immune cells, such as CD4+ T-cells, CD8+ T-cells, and B-cells response, reactive against myelin antigens. Under physiological conditions, the BBB has a protective function, while in the course of MS, it becomes dysfunctional and allows CD4+ T-cells, CD8+ T-cells, and B-cells to infiltrate into the brain [18]. This results in damage to the myelin sheaths surrounding the axons, followed by axonal injury leading to the progression of the patient’s disability [19]. Disruption of the BBB is one of the critical, specifically targeted incidents that initiate MS. The activity of pro-inflammatory cytokines, such as tumor necrosis factor-α (TNF-α) and interferon-γ (INF-γ) leads to the malfunction of this protective barrier [20]. The main hallmark of MS is demyelination of the cerebral white matter. Spinal cord and cerebral cortex demyelination also contribute to permanent neurological disability in this disorder [21]. Destruction of the myelin sheaths is a consequence of an autoimmune process directed against a putative myelin autoantigen mediated by CD4+ T-cells, especially T helper (Th) 1 cells producing IFN-γ and Th17 producing interleukin (IL)-17 [15,22].

The specific signs of MS are gadolinium-enhancing inflammatory lesions (plaques) mainly in the white matter of the CNS, visible on magnetic resonance imaging (MRI), which must be characterized by dissemination in time (DIT) and dissemination in space (DIS), according to the McDonald’s criteria. Radiological and laboratory tests are performed to confirm the diagnosis [23]. The clinical manifestations of MS are individually differentiated neurological symptoms, including, e.g., impaired vision and sensation deficits [16]. A common MS symptom that results from the central nerve injury is neuropathic pain. It leads to sleep disorders, anxiety, and depression and contributes to a decrease in the quality of life and socioeconomic issues. It manifests, e.g., by hyperalgesia, spontaneous pain, and allodynia [24]. It occurs in up to 75% of patients and its treatment is often unsatisfactory for them, even though pain treatment accounts for as much as 30% of drugs used to alleviate the symptoms of MS [25]. Damage to the white and gray matter of the CNS resulting from inflammation and demyelination leads not only to physical disability but also to neuropsychiatric symptoms in MS patients. These signs are observed even in the early phase of the disease and constitute an important component of the diagnostic process and the therapy. The category of neuropsychiatric symptoms in MS covers a broad range of features that distinguishes between cognitive disorders and disturbances impacting mood, behavior, and affect [26,27].

Other symptoms include urinary tract dysfunction, intestinal disorders, spasticity, sexual dysfunctions, dizziness, and fatigue [28]. Non-specific signs such as migraine (alone or in combination with other disorders), fibromyalgia, or non-specific/non-localizing neurologic symptoms with abnormal MRI may lead to misdiagnosis. The 2017 McDonald’s criteria emphasize the role of paraclinical testing on the way to distinguish MS from other diseases with overlapping symptoms, e.g., neuromyelitis optica spectrum disorders (NMOSDs) [29,30]. The main tests used for diagnosis and to support the treatment are cerebrospinal fluid (CSF) analysis in the context of the presence of oligoclonal bands, MRI for active lesions in the brain white matter, and John Cunningham (JC) virus antibody titers [31]. The concept of no evidence of disease activity (NEDA) was introduced to evaluate the efficacy of the treatment used. NEDA generally refers to disease stabilization as evidenced by the lack of clinical relapses, no disease progression as measured by the Expanded Disability Status Scale (EDSS), and the absence of new disease activity on MRI. Recently, an extension of the concept of the NEDA to include brain atrophy and analysis of cognitive function measurement has been proposed [32].

There is no available treatment for MS. Currently approved disease-modifying therapies (DMTs) that act via immunomodulatory/immunosuppressive mechanisms apply to RRMS to reduce relapses frequency and delay disease progression. The DMTs used include interferon-β (INF-β), glatiramer acetate, or natalizumab, which interfere with inflammation. However, recent recommendations point to siponimod and ocrelizumab as drugs that may reduce the progression of SPMS and PPMS, respectively [33,34]. The process of finding the most optimal therapy is highly individualized due to the dynamic and diverse course of the disease. Deciding between choosing more effective but also riskier DMTs in the early stage of MS remains a challenge for clinicians. The methods currently used for the diagnostic and prediction of the DMTs effectiveness have limitations of specificity and sensitivity [35].

## 3. miRNA, Facts, and Expectations

Features such as stability, convenience in extraction from body fluids, and the ability to change the expression profile in the early stages of a specific disease allow miRNAs to be considered as potentially effective diagnostic and prognostic factors [36]. In 2009, Otaegui et al. suggested miRNAs as promising biomarkers in MS and selected those that may be indicators of relapse phase [37]. Regev et al. have already demonstrated that it is a useful tool to discriminate MS patients from healthy controls. Furthermore, 10 particular miRNAs were correlated with EDSS score [38]. The review by Baulina et al. presented the list of miRNAs with altered expression in the course of MS obtained from different sources (CSF, demyelinating plaques, whole blood, plasma, MNCs, T-cells, B-cells), which includes, e.g., miR-155, miR-146a, miR-181c, miR-326, miR-346, miR-17, miR-320a, miR-34a, miR-340, miR-132, and their predicted target genes [39]. However, the functions of miRNAs in the context of MS pathophysiology remain largely unknown.

The transcription of genes encoding miRNAs takes place with the participation of RNA II polymerase, leading to the formation of long primary miRNAs (pri-miRNAs) [40]. Subsequently, the microprocessor complex of RNAse III type Drosha enzyme and DiGeorge syndrome critical region 8 (DCGR8) protein cleaves pri-miRNA in the nucleus into hairpin sequences of precursor miRNAs (pre-miRNA) [41]. Here, the nuclear phase of the processes ends, and the pre-miRNA is transported by the Exportin-5 protein to the cytoplasm. There, pre-miRNA is cleavage by RNase III type Dicer forming an RNA duplex with a passenger strand and a guide strand [42]. This molecule contains mature miRNA sequences that can form an integral compound within the core of a multiprotein RNA-induced Silencing Complex (RISC). The core of a mature RISC consists of Argonaute family proteins (Ago) that remove the passenger strand—typically the guide strand is the one with lower thermodynamical stability of 5′ [43].

Single-stranded miRNA is a template for the recognition of complementary sequences known as miRNA response elements (MREs), located mostly within 3′-untranslated regions (3′UTR) of the mRNA transcript. Within the miRNA, there is a so-called “seed” region located at nucleotides 2–8 from the 5′ end that designates a target. miRNAs play a role in a natural mechanism of specific gene silencing after transcription known as RNA interference (RNAi). The mechanism of gene silencing depends on the degree of match with the target with two types of effects occurring in most cases. Perfect complementarity leads to the cleavage of mRNA by the catalytic Ago2 protein and destabilization and degradation of the transcript [11,14,44,45,46]. If the complementarity is non-canonical, which means, not full, it results in translational repression—this type concerns 60% of interactions [47]. The consequences of the miRNA regulatory mechanism are changes in biological processes such as inflammation, proliferation, differentiation, and cells apoptosis [36,37]. Moreover, miRs modify the transcripts of proteins involved in neurogenesis, gliogenesis, and myelin repair. Dysregulation of specific miRNAs expression may underlie neurodegenerative processes and autoimmunity [48].

Many miRNAs circulate in biological fluids, derived from active transport or revealed through damaging processes such as apoptosis. Circulating miRNAs are packed into vesicles called exosomes (10% of circulating miRNAs) or form complexes with proteins (90% of circulating miRNAs) that prevent digestion by RNases. The miRNAs circulating in the blood are much better detectable than intracellular ones [11,49].

A given mRNA can be targeted by multiple miRNAs, as well as a particular miRNA molecule can influence hundreds of mRNA transcripts, since various genes share the same miRNA recognition site. Nevertheless, the biological significance of various targets can be different [45].

## 4. miR-155 and Immune Response

miR-155 is among those miRNAs that are most strongly implicated in autoimmune diseases [13]. It is encoded by the miR-155 host gene (*mir155hg*) that produces miR-155-3p and miR-155-5p forms. It was formerly called the B-cell integration cluster (BIC) and was found to be strongly overexpressed in B-cell-activated lymphomas [50]. Further research demonstrated that miR-155 occurs in human tissues such as the spleen, thymus, liver, lung, and kidney and is specific for hematopoietic cells [51,52]. The available data suggest the high significance of miR-155 for the homeostasis and the immune response. Namely, the alignment of B-cells functions such as antibody differentiation and production, modulation of T-cells, including CD8+, differentiation of CD4+ toward Th1, Th2, Th17, and regulatory T (Treg) cells, regulation of dendritic cells, cytokines, chemokines, and transcription factors stimulation [14,53]. Furthermore, miR-155 increases activation of astrocytes and affects microglia and macrophages, causing pro-inflammatory polarization to M1-like phenotype, and neurotoxicity [54].

miR-155 is a pleiotropic miRNA characterized by a significant association with genes related to the immune response among which are TNF-α and nuclear factor kappa-light-chain-enhancer of activated B cells (NF-κB) transcripts. It is the main promoter of the development of neuroinflammation manifested by glial activation and pro-inflammatory cytokines production by the CNS-resident cells, a process occurring in neurodegenerative disorders [55,56,57]. After activation of toll-like receptors (TLRs), miR-155 modulates inhibitors of inflammation such as suppressor of cytokine signaling 1 (SOCS1), Src homology-2 domain-containing inositol 5-phosphatase 1 (SHIP1), transcription factor enhancer-binding protein beta (C/EBP-beta), interleukin 13 receptor, and alpha 1 (IL13Rα1) within macrophages/microglia [58,59,60,61]. The pro-inflammatory action of miR-155 in microglia may also be activated by the transcriptional factor p53. In this case, the target of miR-155 becomes the transcription factor c-Maf that normally induces anti-inflammatory processes in the immune cells. The effect of this action is the promotion of inflammation [62].

Overexpression of miR-155 has been observed in a range of samples, including resident brain cells, active brain lesions, as well as blood cells from MS patients [15]. Junker et al. demonstrated that miR-155 was up-regulated (fold change = 11.9; *p* < 0.01) in active white matter lesions compared with healthy controls [63]. Paraboschi et al. carried out a study that investigated the expression levels of 22 miRNAs associated with immunity and located in the peripheral blood mononuclear cells (PBMCs) of RRMS patients in the remission phase compared with healthy controls. As a result, miR-155 was found to be the most up-regulated (fold change = 3.30; *p* = 0.013) indicating its involvement in the regulation of MS pathophysiology [64]. The study carried out on an experimental autoimmune encephalomyelitis (EAE), an animal model for MS, revealed overexpression in CD4+ T-cells from the spleen, lymph nodes, and CNS compared with controls and thus showed that miR-155 confers susceptibility to EAE [65]. It has been found that miR-155 is one of the most crucial miRNAs in MS as it regulates MS risk genes *PIK3R1* and *PIK3CA* and correlates with severity of the disease [49]. These genes encode p85-α and p110-α proteins, members of phosphoinositide 3-kinase (PI3K) family [66,67]. Abnormalities in PI3K contribute to, e.g., cancer development, neurological and immunological dysfunctions, dendritic cells functioning, EAE pathogenesis and demyelination in MS [49]. The results of further research in the context of MS and EAE confirmed the association of miR-155 with these disease states. Up-regulation of miR-155 might indicate severe condition course and poor prognosis in MS patients [68,69,70]. The pro-inflammatory effect of miR-155 affects microglia activation, phagocytosis of myelin by macrophages [63], differentiation of T-cells towards Th1 or Th2 [71], contribution to increased permeability of the BBB, and infiltration of peripheral immune cells [72]. In recent studies, attempts have been made to check potential links between the miR-155 expression level and depression, cognitive functions, and neuropathic pain [55,73,74,75,76]. As miR-155 is generally associated with inflammation, it is likely to be involved in various pathophysiological processes and symptoms of MS.

## 5. miR-155 and Blood–Brain Barrier

The BBB vascularizes the CNS; selects the movement of leukocytes, pathogens, nutrients, and proteins from blood to the brain; and takes part in cell-to-cell interactions. It is a unique semi-permeable structure of brain capillaries, formed by cerebral endothelial cells (ECs), pericytes, and astrocytes. ECs are essential for the integrity of BBB and cerebral homeostasis. Cellular junctions consist of selective complexes, mainly tight junctions (TJs) and adherens junctions (AJs), composed of smaller subunits of transmembrane proteins, such as occludin, claudins, junctional adhesion molecules, zonula occludens, cadherins, and catenins [16,77,78].

Although the mechanism of BBB disassembly is not fully elucidated, it is believed that the pro-inflammatory cytokines contribute to this phenomenon, among others, at the transcriptional stage and/or during post-transcriptional regulation of gene expression. Moreover, it is known that vascular endothelial cells are abundant in miRNAs [79].

**Figure 1:**
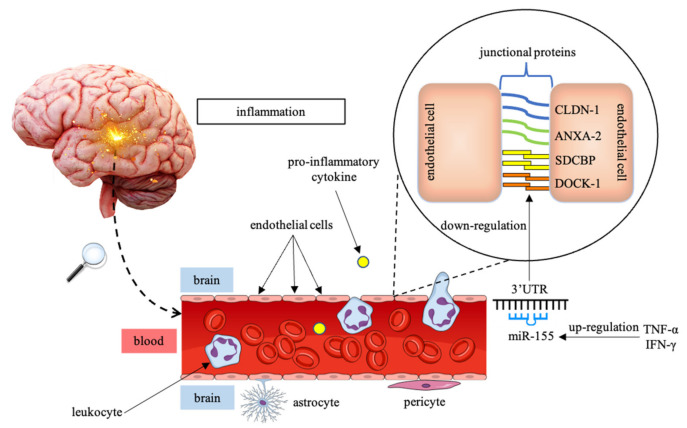
Operation of miR-155 in disruption of the BBB under inflammatory conditions. miR-155 is up-regulated due to pro-inflammatory cytokines activity, such as TNF-α and IFN-γ. Overexpression of miR-155 enhances an inflammatory effect contributing indirectly to BBB permeability via down-regulation of junctional proteins between endothelial cells: CLDN-1, ANXA-2, SDCBP, and DOCK-1. Abbreviations: BBB—blood–brain barrier; TNF-α—tumor necrosis factor-α; IFN-γ—interferon-γ; CLDN-1—claudin-1; ANXA-2—annexin-2; SDCBP—syntenin-1, DOCK-1—dedicator of cytokinesis-1.

Lopez-Ramirez et al. confirmed that the activity of miR-155 affects the function of BBB at the neurovascular unit (NVU) components in both brain of MS patients and spinal cord of EAE mouse [72,80]. The NVU is a comparatively new concept in neuroscience, defined as a multicellular structure composed of neurons, perivascular astrocytes, microglia, pericytes, BBB ECs, and the basement membrane. The NVU is the fundamental driver of neurovascular coupling that plays an essential role in all stages of BBB development and maintenance. The NVU can act in a synchronized, functional way and regulate cerebral blood flow maintaining brain homeostasis. Damage to NVU components is connected with reduction in permeability as well as selectivity of BBB [80]. miR-155 was demonstrated to be up-regulated in mice, and loss of this regulator caused higher tightness of BBB within the neuroinflammatory loci. Furthermore, the expression level of miR-155 is increased in brain endothelium in active MS lesions, and the analysis performed on cell culture of brain endothelial cells (BECs) confirmed that miR-155 overexpression is stimulated by pro-inflammatory cytokines, TNF-α, and IFN-γ. Therefore, it is suggested that brain endothelial miR-155 is induced by inflammation and subsequently stimulates an early inflammatory effect at the NVU. The up-regulation of miR-155 influenced BBB permeability mimicking the alternations that were induced by cytokines. The mechanism by which miR-155 exacerbated BBB disruption relies on down-regulation of junctional proteins, significant for BBB integrity: claudin-1 (CLDN-1), annexin-2 (ANXA-2), syntenin-1 (SDCBP), and dedicator of cytokinesis-1 (DOCK-1) (Figure 1). CLDN-1 and ANXA-3 belong to interendothelial junctional complex molecules, while SDCBP and DOCK-1 constitute focal adhesion components. Thus, overexpression of miR-155 indirectly contributes to BBB malfunction by negatively regulating elements of the cell-to-cell and cell-to-matrix adhesion pathways [72,81].

The following study conducted on the cytokine-stimulated cultured human brain endothelium revealed the contribution of miR-155 up-regulation to increased adhesion of monocytes and T-cells to the BBB during neuroinflammation. It has been suggested that miR-155 acts via modulation of endothelial adhesion molecules, vascular cell adhesion molecule 1 (VCAM1), and intercellular adhesion molecule 1 (ICAM1) [79]. Noteworthy is the study carried out on an animal model of stroke, which supports brain endothelial miR-155 as an important player in the process of neuroinflammation and disruption of the BBB integrity. Additionally, it presents a protective effect of anti-microRNA-155 in vivo [82]. In contrast, increased expression of other miRNAs, miR-98 and let-7 in vitro and in vivo decreased leukocyte adhesion to and infiltration across ECs, inhibited pro-inflammatory cytokines, and supported BBB during neuroinflammation [83]. It is also worth mentioning that the importance of miR-155 modulation in the context of BBB disruption has been confirmed in several studies associated with post-ischemic endothelial injury and recovery [82,84,85].

Interestingly, the lipopolysaccharide (LPS)-activated layer of epithelial cells composing the blood-CSF barrier (BCSFB) called choroid plexus epithelium (CPE) has been shown to release exosomes into the CSF during inflammation. These extracellular vesicles (EVs) containing miR-155 reach the brain parenchyma and stimulate cytokines such as TNF, IL-1, and IL-6 giving rise to inflammation. Although the BBB function is undeniably important in the blood-to-brain signaling and the production of EVs by ECs has not been excluded, it has been emphasized that ECs manifest low vesicle transport activity. Moreover, the BBB integrity was not disrupted by LPS activity; this fact is argued against the role of BBB permeability in the inflammation-induced EVs increase in the CSF [86].

## 6. miR-155 and Demyelination

The main cells that are responsible for myelin production in the CNS are oligodendrocytes differentiated from the oligodendrocyte precursor cells (OPCs). The phenomenon of myelin loss occurs when the differentiation process is compromised, e.g., by the resident innate immune cells of the CNS, involved in antigen presentation and secretion of pro-inflammatory factors—microglia, leading to the destruction of the myelin sheath or/and oligodendrocytes [87].

miR-155 contributes to the activation of the microglia, which produces pro-inflammatory factors including TNF-α, IL-1b, IL-6, interferon-inducible protein 10 (IP-10), macrophage inflammatory protein-1α (MIP-1α), monocyte chemoattractant protein-1 (MCP-1), and nitric oxide (NO) [16]. Interestingly, miR-155 has been shown to promote the polarization of astrocytes towards their activated neurotoxic form A1 [15]. Astrocytes can adopt at least two different reactive phenotypes (A1 and A2) in response to CNS insult. The former type is induced as a result of CNS disease, acute injury, and LPS-induced neuroinflammation and can lead to the death of neurons and oligodendrocytes. In contrast, astrocytes A2 are induced by ischemia and may act protective by upregulating neurotrophic factors. Under normal conditions, astrocytes contribute to neuronal survival, however, subtype A1 has lost this function. It has been revealed that activated microglia might induce the astrocytes’ transformation into the A1 form by releasing interleukin 1-α (IL-1α), TNF, and complement 1, and subcomponent q (C1q). The enhanced level of A1 astrocytes was reported in different human neurodegenerative diseases, including MS [15,16]. The level of the transmembrane CD47 protein, which reduces microglial activation, is decreased by overexpression of miR-155 in brain active lesions. CD47 acts by inducing a “do not eat me” signal on cells adjacent to the microglia. Overexpression of miR-155 is involved in macrophages activation by decreasing CD47 expression in astrocytes and oligodendrocytes, and also causes myelin phagocytosis by macrophages, affecting them directly [63].

In one study, Mycko et al. analyzed the alternations of miR-155, miR-301a, and miR-21 expression profiles in the context of CD4+ T-cells. The research was conducted on the animal model of the disease, which was induced by immunization with the CNS antigen, myelin-oligodendrocyte glycoprotein (MOG) 35–55. The results demonstrated that all miRs studied, including miR-155, were significantly elevated in vitro and in vivo. Interestingly, in this study, only miR-301a was found to be involved in Th17 differentiation and the pathogenesis of demyelination via Protein Inhibitor of Activated STAT 3 (PIAS3) molecule (affecting IL-6/23–STAT3 pathway) as a direct target in CD4+ T-cells. Inhibition of miR-155 and miR-21 by specific inhibitors, called antagomirs (anti-miRs) caused down-regulation of IFN-γ and up-regulation of IL-4 secretion [88]. However, the crucial role of miR-155 in Th17 cells has been demonstrated in the context of autoimmune inflammation during EAE via the action involving transcription factor Ets1 and the clinically relevant IL-23-IL-23R pathway [89]. Another study demonstrated that miR-155-3p targets two genes in CD4+ T-cells: *Dnaja2* and *Dnajb1*, which are related to the down-regulation of Th17 lymphocytes and their infiltration into the brain. It has been suggested that miR-155-3p operates in the process of autoimmune demyelination with selective expression during EAE [22]. The results of recent studies based on the EAE model induced by cuprizone (a copper chelator molecule that affects oligodendrocytes) confirmed that decreasing expression of miR-155-3p can prevent microglia activation, myelin damage, and MS progression [90,91]. Analysis of miR-155 expression carried out on a mouse model allowed revealing the effect of the probiotic bacteria, *Lactobacillus casei,* on the recovery of demyelinated animals by regulating the immune response [92]. Another research conducted on the cuprizone-induced demyelination model evaluated the miRNAs expression and predicted their target genes according to the micro-RNA database (miRDB). miR-155-5p was among the upregulated miRNAs with fold change ≥ 1.5, compared with the control group. Prediction of target genes could reveal connections between miRs modulation and the cascade of processes during demyelination induced by cuprizone administration. Among the targets was a transcription factor mothers against decapentaplegic homolog 2 (SMAD2), mediating the anti-inflammatory effects of transforming growth factor-β (TGF-β), the pathway of which was previously considered one of the regulatory demyelination pathways. Up-regulated miR-155-5p may play a role in the mechanism of demyelination through a suppressive effect on Smad molecular cascades, leading to upregulation of the Nogo receptor (NgR) present on axons to inhibit neurite outgrowth [93].

## 7. miR-155 and Neuropathic Pain

Emerging data suggest neuropathic pain as a neuro-immune dysfunction with increased activation of the immune system. miR-155 has a well-studied influence on inflammatory factors such as IL-1β, IL-6, TNF-α, NF-κB, and p38 mitogen-activated protein kinase (MAPK), associated with neuropathic pain, [94,95,96]. miR-155 was demonstrated to be up-regulated in the prefrontal cortex of mice with inflammatory pain [97]. A rat model of neuropathic pain, chronic constrictive injury (CCI), allowed noting increased expression level of miR-155 in spinal microglia, while its inhibition alleviated neuropathic pain and neuroinflammation. It has been investigated that suppression of miR-155 caused inhibition of NF-κB and p38 MAPK activation by mediating a negative regulator of inflammation, SOCS1 [98]. Moreover, miR-155 has been demonstrated to modulate an inflammation-related serum and glucocorticoid regulated protein kinase 3 (SGK3) in neuropathic pain rats. Down-regulation of miR-155 and stimulation of SGK3 expression resulted in alleviation of the nerve pain and lower pain threshold [99]. Both miR-155 and miR-124a are predicted to target the histone deacetylase sirtuin 1 (SIRT1). This interaction enhances the development of anti-inflammatory Tregs by increasing the expression of transcription factor forkhead box-p3 (Foxp3), which is a key regulator of Tregs differentiation. Promotion of Tregs leads to alleviation of pain hypersensitivity suggesting a possible role of these cells in the mitigation of the pain-promoting inflammatory response [100].

Another gene regulated by miR-155 is NADPH oxidase (NOX2), an inducer of reactive oxygen species (ROS) in macrophages/microglia [101]. NOX2 regulation allows manipulating the proportion of pro-inflammatory (M1) and anti-inflammatory (M2) phenotypes of macrophages/microglia [102,103]. Increased expression of this enzyme has been reported after spinal cord injury. It contributes to neuropathic pain development via M1 microglia/macrophages activation and inflammatory-related cascades [104]. Inhibition of NOX2 reduces ROS production, stimulates microglia differentiation towards M2, increases expression of anti-inflammatory cytokine IL-10, and decreases expression of pro-inflammatory factors, including miR-155 [100,105]. It has been demonstrated that miR-155 expression, which is elevated in the spinal cord injury (SCI) model, is lower in NOX2−/− mice and it may be controlled by IL-10 [101,106]. Microglial miR-155 has recently been investigated as an important player in neuropathic pain development by engaging in M1/M2 polarization switch. The research was conducted on the BV-2 type microglial cells model, and the M1/M2 polarization was induced by LPS/IL-4. The neuropathic pain model was created by generating spinal nerve ligation in rats. The elevated miR-155 expression has been noted in the M1 microglia phenotype and lowered in M2. Suppression of miR-155 reduces the secretion of IL-1β and TNF-α and alleviates neuropathic pain by stimulating the switch from pro-inflammatory M1 to anti-inflammatory M2 polarization [76]. Another study investigated the role of miR-155 in a disorder manifested by a chronic neuropathic pain associated with demyelination—trigeminal neuralgia (TN). It was demonstrated that miR-155-5p is overexpressed in the serum of TN patients and inhibits nuclear factor erythroid 2-related factor 2 (Nrf2) expression, which was previously shown as a neuroprotective factor in diabetic peripheral neuropathy [107,108].

## 8. miR-155 and Neuropsychiatric Symptoms

Damage to the BBB, loss of myelin and axons, synaptic dysfunction, and dysregulated neurotransmitter production due to acute inflammation result in brain atrophy and progressive cognitive impairment [48]. Depression and anxiety appear to worsen memory, information processing speed, and daily functioning in people with MS [26].

It is estimated that the prevalence of depressive disorders among people with MS is up to 50%, which means 2–3 times higher than in the general population. Besides anxiety and stressors related to the presence of the disease, pathophysiological mechanisms such as brain atrophy, activation of pro-inflammatory cytokines, astrocytes and microglia, and lesion burden contribute to depressive disorders [109]. It is believed that mood disorders development results from complex mechanisms related to genetic predisposition and environmental factors. It is suggested that epigenetic mechanisms have a great role in pathogenesis. Research results revealed alterations in miRNAs expression levels, including miR-155, targeting neurotrophic factors (brain-derived neurotrophic factor, BDNF, TGF-β) and inflammatory mechanisms in patients with depressive disorders [73,110,111]. In the CSF and blood samples of people suffering from a major depressive disorder (MDD), the expression of miR-155 was elevated [112,113].

Fonken et al. probably were the first to demonstrate the effect of experimental modulation of miR-155 expression on anxiety- and depressive-like behaviors in an animal model. Mentioned behaviors, as well as sensorimotor function and social behavior, have been assessed in miR-155 knockout and wild-type mice. Additionally, the expression of genes related to inflammatory and neurotrophic factors was evaluated in the hippocampus, since changes in this structure are associated with mood disorders. In mice with the miR-155 deletion, a reduction in anxiety and depressive behaviors was observed, however, no effects on memory, learning, social behavior, and sensorimotor skills were found. This demonstrates the specificity of miR-155 activity. Analysis of changes in miR-155 expression levels revealed that inactivation of this factor reduces inflammation in the hippocampus by decreasing the expression of genes encoding the pro-inflammatory cytokines, IL-6, and TNF-α. Furthermore, mRNA encoding a neurotrophic cytokine that promotes neurogenesis in several regions in the brain, ciliary neurotrophic factor (CNTF), was increased in the hippocampi of female mice lacking miR-155 [73]. The study conducted on mice cell lines revealed a possible role of miR-155 in the context of treatment-resistant depression. miR-155 down-regulated SOCS1, leading to the activation of microglia that contributes to inflammatory injury of hippocampal neurons [114]. In contrast, the correlation analysis of miR-155 expression in RR-MS patients (in the remission phase) with the Beck Depression Index (BDI) scale and with Montreal Cognitive Assessment (MoCA) values did not reveal to be statistically significant [74].

Chronic stress occurring simultaneously with EAE has been shown to exacerbate the clinical manifestations of the disease. Furthermore, deep sequencing revealed changes in the expression of miRNAs that are important in human MS. Both miR-155 and another epigenetic hallmark of MS, miR-146a, were up-regulated in the lumbar spinal cord during EAE, as well as a result of stress exposure. It has been suggested that stress may synergistically exacerbate the severity of EAE through changes in the expression of epigenetic factors. This supports the role of MS-associated stress in disease worsening and the significance of miR-155 as a biomarker for monitoring disease progression [115].

The study conducted on pediatric multiple sclerosis (PedMS) patients demonstrated that the expression panel of 11 miRNAs (miR-181a-5p, miR-99b-5p, miR-25-3p, miR-148b-3p, miR-125a-5p, miR-185-5p, miR-182-5p, miR-320a, miR-652-3p, miR-942-5 and miR-221-3p; out of 13 primarily selected), derived from the peripheral blood samples, were associated with genes such as *NTNG2*, *BST1*, *STAB1*, and *SPTB*, possibly related to cognitive abilities. The selection of miRs for the study was based on significant differences in their expression between PedMS patients and healthy controls. These results may be a step towards finding reliable molecular indicators of cognitive impairment in MS and effective monitoring disease progression, however, the authors emphasized the limitations of this research due to small study group (n = 19) or inability to compare results with adult MS patients [116]. To date, no studies referred to cognitive impairment in adult MS patients have been published. As such, Varma-Doyle et al. provided a scoping review of miRs that are dysregulated in both MS and in cognitive disorders, including dementia, to find the overlapping ones and explore their possible role in MS-associated cognitive impairment. The miRs analyzed were found to be capable of modulating proteins with neuroprotective and/or neurodegenerative properties. Of them, miR-155, miR-15, miR-132, miR-138, and let-7 contributed to the elevation of neurodegenerative proteins, e.g., SRY-Box transcription factor 4 (SOX4). miR-155 was one of the dysregulated miRNAs overlapping MS and dementia via association with neuroinflammation and activation of microglia. This study further identified overlapping dysregulated miRs affecting various processes, including neuronal repair and remyelination, apoptosis, glutamate toxicity, and amyloid deposition that might play a role in dementia in MS patients. The need for further research to investigate the role of these miRs specifically in the context of cognitive changes in MS has been emphasized [48].

Up-regulated miR-155 located on the triplicated chromosome 21 has been shown to play a key role in the development of dementia in individuals with Down syndrome [117]. In Alzheimer’s Disease (AD), miR-155 contributed to the regulation of cognitive functions by participating in neuroinflammatory processes, which was demonstrated in AD rats with memory impairment [118]. In contrast, the analysis of serum exosomal miRNA in patients with dementia did not reveal a significant change in expression of miR-155 compared with controls, pointing to another inflammation-related miR—miR-223 as a valuable diagnostic parameter. The levels of miR-223 were significantly decreased and negatively correlated with inflammatory markers. This result was supported by dementia severity assessment tools and magnetic resonance spectroscopy [119].

## 9. Future Perspectives

Following the databases, there is strong evidence that miR-155 is of interest to researchers and clinicians as an effective biomarker candidate for monitoring treatment efficacy as well as a therapeutic target, considering the results of studies in which anti-miRs are tested.

Presently, there is an ongoing clinical study that aims to investigate the correlation between miR-155 and miR-150 with different MS phenotypes, disability status, and the patient demographic data [120,121]. Worth attention are the actions of the American company miRagen Therapeutics that developed a drug cobomarsen (MRG-106) designed to inhibit the activity of miR-155 in patients with cutaneous T-cell lymphoma (CTCL) and mycosis fungoides (MF) subtype [122,123]. Clinically, there were no serious adverse reactions attributed to these anti-miR-155 molecules, as well as no evidence of immunosuppression over nearly 2 years [124]. miRagen Therapeutics has also a completed preclinical trial for drug MRG-107, targeting miR-155 designed for the treatment of amyotrophic lateral sclerosis (ALS). What is more, the biotech company DiamiR provided miRNA diagnostic blood tests used in studies on biomarkers for detection and differentiation of neurodegenerative diseases [7,125]. The drug discovery development of miRNA and their status in clinical trials have been recently reviewed by Chakraborty et al., who placed great emphasis on the essence of conducting more clinical trials and supporting future perspectives in this field [126].

Increasing understanding and exploration of miR-155 mechanisms may contribute to the initiation of further studies on the application of these molecules in clinical practice. Currently, numerous pharmaceutical companies are working on the development of miRNAs as novel biomarkers and therapeutic targets of various diseases, including MS.

## 10. Conclusions

Abnormalities in the pathogenesis of MS may originate from genetic, exogenous, and environmental factors. The search for relatively simple, specific, and sensitive methods of diagnosing and monitoring the course of MS remains a challenge. The results of the analyses performed so far indicate that miRNAs may represent a sensitive and specific determinant for this condition stage. Of them, miR-155 has a great potential to be implemented into clinical trials toward qualifying it as a biomarker due to its extensive impact on inflammatory molecular pathways, detectable alternations in expression levels, and ability to be inhibited by specific anti-miRs. As suggested by the research findings, miR-155 affects key components of the BBB under inflammatory conditions contributing to its disruption and has a huge impact on demyelination by stimulating various pro-inflammatory factors. Emerging studies indicate a significant role of miR-155 in regulating the development of neuropathic pain in general, which may be a great hint for further research specifically on MS. This epigenetic factor is also suggested to be an important player in complex processes involved in neuropsychiatric symptoms development appearing as a serious problem affecting the quality of life in MS patients. It may be considered as a possibly good candidate for further research toward innovative therapeutic targets because of the ability to obtain an effect on a wide range of pathophysiological factors simultaneously. However, targeting multiple transcripts may be also restrictive due to the limited specificity of the action. miR-155 has been already evaluated as a general regulator of inflammation. Successive exploration of its specific role, in particular, pathophysiological processes in MS, is a promising path toward a better understanding of the mechanisms of this complex disease and more effectively diagnosing, treating, and monitoring it.

## Data Availability

No new data were created or analyzed in this study. Data sharing is not applicable to this article.
